# Investigating the clinical use of structured light plethysmography to assess lung function in children with neuromuscular disorders

**DOI:** 10.1371/journal.pone.0221207

**Published:** 2019-08-26

**Authors:** Deborah Fleck, Chistopher Curry, Kate Donnan, Orla Logue, Kathryn Graham, Kate Jackson, Karen Keown, John Winder, Michael D. Shields, Ciara M. Hughes

**Affiliations:** 1 Nursing and Health Research Institute, School of Health Sciences, Ulster University, Belfast, Northern Ireland, United Kingdom; 2 Centre for Experimental Medicine, Queen’s University Belfast, Belfast, Northern Ireland, United Kingdom; 3 Royal Belfast Hospital for Sick Children, Belfast Health & Social Care Trust, Belfast, Northern Ireland, United Kingdom; Vanderbilt University Medical Center, UNITED STATES

## Abstract

**Background:**

Children and young people with neuromuscular disorders (NMD), such as Duchenne Muscular Dystrophy (DMD), develop progressive respiratory muscles weakness and pulmonary restriction. Pulmonary function monitoring of the decline in lung function allows for timely intervention with cough assist techniques and nocturnal non-invasive ventilation (NIV). NMD may find the measurement of lung function difficult using current techniques. Structured Light Plethysmography (SLP) has been proposed as a novel, non-contact, self-calibrating, non-invasive method of assessing lung function. The overarching aim of this study was to investigate the use of SLP as a novel method for monitoring respiratory function in children with neuromuscular disease.

**Methods:**

SLP thoraco-abdominal (TA) displacement was correlated with forced vital capacity measurements recorded by spirometry and the repeatability of the measurements with both methods examined. SLP tidal breathing parameters were investigated to assess the range and repeatability of regional right and left side TA displacement and rib cage and abdominal wall displacement.

**Results:**

The comparison of the FVC measured with SLP and with spirometry, while having good correlation (R = 0.78) had poor measurement agreement (95% limits of agreement: -1.2 to 1.2L) The mean relative contribution of right and left TA displacement in healthy controls was 50:50 with a narrow range. Repeatability of this measure with SLP was found to be good in healthy controls and moderate in NMD children with/without scoliosis but with a wider range. The majority of the control group displayed a predominant rib cage displacement during tidal breathing and those who displayed predominant abdominal wall displacement showed displacement of both regions close to 50:50 with similar results for the rib cage and abdomen. In comparison, children with NMD have a more variable contribution for all of these parameters. In addition, SLP was able to detect a reduction in abdominal contribution to TA displacement with age in the DMD group and detect paradoxical breathing in children with NMD. Using SLP tracings during tidal breathing we were able to identify three specific patterns of breathing amongst healthy individuals and in children with NMD.

**Conclusions:**

SLP is a novel method for measuring lung function that requires limited patient cooperation and may be especially useful in children with neuromuscular disorders. Measuring the relative contributions of the right and left chest wall and chest versus abdominal movements allows a more detailed assessment.

## Introduction

Children and young people with neuromuscular disorders (NMD), such as Duchenne Muscular Dystrophy (DMD) and Spinal Muscle Atrophy (SMA), develop progressive weakness of the respiratory muscles effectively leading to restrictive pulmonary disease [1]. Reduced ability to expand the thoraco-abdominal wall (TA) predisposes these patients to recurrent chest infections, nocturnal hypoventilation and ultimately respiratory failure, which is the most common cause of morbidity and mortality [2]. It was previously estimated that 55–90% of DMD patients, without interventions, die from respiratory failure between 16 and 19 years of age [3,4]. Pulmonary function testing is crucial to monitor the declining lung function and indicate when interventions such as cough assist techniques and nocturnal non-invasive ventilation (NIV) should be initiated. These non-invasive interventions prolong the survival and improve the quality of life of these patients.

Currently it is recommended that most children with NMD have their lung function monitored by spirometry (Forced Vital Capacity, FVC), peak cough flow measurement, tests of respiratory muscle strength (maximal inspiratory and expiratory pressures) and overnight sleep records measuring O_2_ saturation and either end tidal or transcutaneous CO_2_ [1]. These provide a global assessment of lung function and do not distinguish between the respiratory muscles involved [5]. Of these, spirometry (FVC) measured with pneumotachography is the most useful and readily available technique used to measure lung function to allow monitoring of disease. In neuromuscular disease there is often no intrinsic lung disorder and hence the forced vital capacity (FVC) and peak cough flow (PCF) actually reflect respiratory muscle strength [6].

There are many difficulties surrounding the measurement of lung function in children with NMD using current techniques. Children with NMD may have pronounced facial muscle weakness making it more difficult for them to maintain a tight seal around the mouth piece when carrying out spirometry [7]. Likewise, some children with NMD, especially DMD, present with autistic features and therefore find it difficult to fully understand how to use the equipment and cooperate with the operator [8]. Another major limitation of spirometry is that it has a poor sensitivity with regards to detecting moderate inspiratory muscle weakness as a reduction in VC is not specific for this condition [9]. In addition, height and age are normally used to predict expected lung function values in children and young people. Measuring the height of a child with NMD can be unreliable due to scoliosis as well as impractical as many of these children are wheelchair bound. This has led to alternative methods for estimating height such as the use of arm span and ulnar bone length neither of which is an accurate replacement for height [10]. Current methods for measuring pulmonary function cannot identify levels of recruitment of different regions of the lungs during breathing. Pulmonary restriction in NMD is further exacerbated by the mechanical effects of progressive scoliosis. An indication of the relative contribution of the right and left sides of the thoraco-abdominal wall (TA) in these patients could be clinically useful for guiding the effectiveness of interventions such as non-invasive ventilation (NIV) therapy and cough assist techniques. It is not known whether optimising lung expansion on both right and left sides using NIV would be beneficial in patients with NMD and scoliosis.

The effect of NMD on the respiratory muscles differs between specific diseases, for example, in children with spinal muscular atrophy (SMA), the diaphragm is the primary muscle for breathing. There is a lack of opposition of the weak intercostal muscles against the function of the diaphragm and the lower abdomen will be pushed out as the diaphragm is pushed down resulting in paradoxical breathing. As a result of progressive muscle weakness, these children will have chest wall deformities including, a bell-shaped chest and pectus excavatum, and this will ultimately make it more difficult for them to expand their TA fully [11]. On the other hand, in children with DMD, the diaphragm is initially affected more than the intercostal muscles which means the abdomen will move inwards rather than outwards during inspiration when the rib cage moves out, again resulting in paradoxical breathing [12]. Current pulmonary function tests cannot characterise the relative contribution of these regions (rib cage and abdominal expansion) of the TA to breathing which would be useful for guiding therapy (e.g. NIV) in these patients where the aim is to optimise TA expansion.

**Fig 1:**
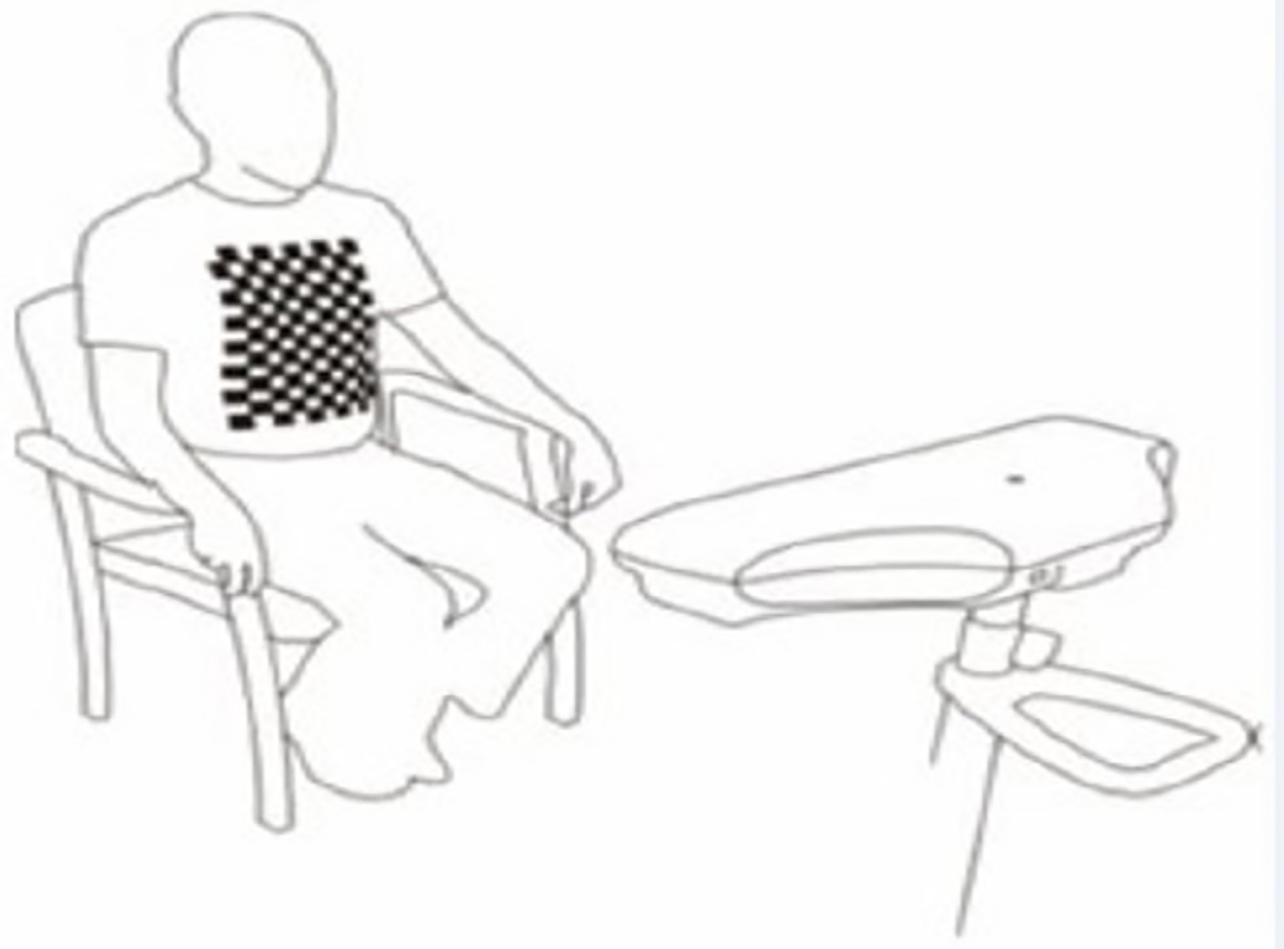
Structured light plethysmography in clinical use (printed with permission from Pneumacare).

Structured Light Plethysmography (SLP) has been proposed as a novel, non-contact, self-calibrating, non-invasive method of assessing lung function [13,14]. This technique assesses changes in lung volume during respiration by measuring the displacement of the TA during quiet (tidal) breathing. A light is projected in a grid/checkerboard pattern onto the patient’s TA as shown in Fig 1.

Data recorded by SLP (following an FVC manoeuvre) provided a numerical value (arbitrary units “estimated litres”) which had previously been shown to correlate well with the forced lung function parameter (FVC) recorded by spirometry [14] however; repeatability issues may have prevented its more widespread use. Recent studies have shown that SLP tidal breathing parameters including breath by breath and averaged respiratory rate (RR), inspiratory time (tI), expiratory time (tE), total breath time (tTot), ratios (tI/tE, tI/tTot) and IE50 (inspiratory to expiratory flow measured at 50% of tidal volume) have a high degree of repeatability [15,16]. SLP measurements could allow TA expansion to be objectively measured in children who are unable to generate reproducible spirometry readings [14]. In addition, and importantly, SLP has the ability to measure displacement of different regions of the TA during breathing. Regional TA displacement could be important for optimising NIV and cough assist equipment settings. It may be beneficial to ensure that NIV or cough assist settings are optimally moving both lungs in those with scoliosis and the TA wall in those children who are predominantly using the diaphragm for breathing. However, little is known about the range of regional TA displacement (rib cage and abdomen; left and right rib cage expansion) during breathing or the repeatability of measuring these with SLP.

The overarching aim of this study was to investigate the use of SLP in a real clinical environment as a novel method for monitoring respiratory function in children with neuromuscular disease. Firstly, SLP TA displacement was correlated with forced vital capacity measurements recorded by spirometry and the repeatability of the measurements with SLP was examined (Study 1). Secondly, SLP tidal breathing parameters were investigated to assess the range and repeatability of regional right and left side TA displacement and rib cage and abdominal wall displacement (Study 2).

## Methods

Patients recruited into both studies were children and young people (aged 5 to 18 years) with neuromuscular disease attending the Respiratory Neuromuscular Clinic in the Royal Belfast Hospital for Sick Children (RBHSC). Patient demographics for Study 1 and Study 2 are shown in Table 1. Approximately half of the patients had Duchenne Muscular Dystrophy with the remaining participants diagnosed with a variety of other NMD disorders. In addition, a group of healthy adult volunteers without a history of respiratory or neuromuscular disease (research team members, hospital staff and medical students) helped the research team to learn and develop the SLP technique and their data was subsequently used (with their agreement) for normal control data.

**Table 1:** Patient demographics for study 1 and study 2.

	Study 1	Study 2
**Total (n)**	13	34
**Mean age (± SD)**	12 (± 4.6)	12.8 (±3.37)
**Age range, n (%)**		
**5–12 yr**	8 (62)	16 (47)
**13–15 yr**	1 (8)	10 (29)
**16–21 yr**	4 (30)	8 (24)
**Gender, n (%)**		
**Female**	4 (30.8)	25 (74)
**Male**	9 (69.2)	9 (26)
**Diagnosis, n (%)**		
**DMD**	6 (46)	19 (55)
**Other** (such as SMA, other Dystrophies and congenital Myopathies)	7 (54)	25 (45)
**Scoliosis, n (%)**		
**Yes**	3 (23)	4 (12)
**No**	10 (77)	30 (88)

All patients were fully informed of the procedure, written parental consent was obtained and children and young people with NMD provided written consent or assent. Ethical approval was given for both studies by the Office Research Ethics Committee Northern Ireland (ORECNI).

For SLP measurement during each study the participants were positioned in front of the SLP device (PneumaCare’s Thora-3Di Compact, Cambridgeshire, United Kingdom, www.pneumacare.com) and a white, fitted sports t-shirt was worn over the participant’s light tee-shirt or vest (in the majority) or without their own clothing (in the minority) as preferred. The patient was asked to place their finger on their xiphysternum and the centre of the SLP grid was aligned with this placement at a distance of 1 metre from the SLP projector. A grid, which covered as much of the TA as possible, without ‘bleeding’ of the grid around the sides of the chest or abdomen or over the shoulders, was selected.

### Study 1: Relating vital capacity measurements from SLP with FVC measured with spirometry

Three attempts at SLP derived forced vital capacity (FVC_(SLP)_) was measured according to the manufacturer’s instructions and the best FVC_(SLP)_ was recorded. In order to determine test repeatability, a second set of SLP measurements were made several minutes after the first; the patient and the SLP equipment was moved and then repositioned as described above. In addition, standard spirometry was performed and measured using spirometry (FVC) with a portable MicroLab spirometer (MicroMedicals,CareFusion) according to the British Thoracic Society testing guidelines [17].

### Study 2: Using SLP Tidal Breathing parameters to examine the relative contribution (%) of right side and left side thoraco-abdominal wall and the relative contribution (%) of rib cage and abdominal wall

SLP provides measurements of the relative contribution of the right and left side TA displacement and the rib cage and abdominal wall contribution during tidal breathing (TB). The manufacturer (PneumaCare) recommend 5 minutes of relaxed tidal breathing, however, this was found to be impossible to achieve in some restless children and therefore 1 minute of tidal breathing was measured. After the initial SLP recording, the patient was repositioned to simulate a second clinic visit and similar SLP settings were used to obtain a repeat 1-minute tidal breathing measurement. The outputs comparing relative contribution of the right and left sides of the TA and the rib cage and abdominal wall were analysed.

### Tidal breathing patterns

The tidal breathing pattern measured with SLP was analysed for each participant to ascertain if there was a pattern unique to children with NMD.

The tidal breathing pattern was acquired for both participants and controls and line graphs produced with the TA displacement (the displacement is presented in arbitrary SLP system units) plotted against time. The TA displacement was sampled by SLP at a rate of 30 data points per second (each data point separated by 0.03333 seconds). The data was background corrected for signal drift related to underlying patient movement using a 3 point cubic spline fitting algorithm (MagicPlotPro V2.5.1, https://magicplot.com). A section of 10 tidal breaths, from the full the 1-minute recording period, was visually selected by a respiratory physiologist where the breathing was judged as relaxed and free from subject motion artefact. Three types of TA displacement pattern were identified and labelled as follows. Pattern 1: regular amplitude and frequency, Pattern 2: regular amplitude and frequency with extended expiratory phase, Pattern 3: irregular amplitude and frequency. Each participant’s breathing pattern was presented to 4 of the researchers, (a consultant respiratory clinician, a respiratory physiologist, a biomedical scientist and a clinical scientist). Each pattern was labelled 1, 2 or 3 by each observer and if there was discrepancy amongst the observers, this was resolved by discussion and agreement.

### Statistical analysis

Normally distributed data was described as mean (standard deviation, SD) and skewed data as median (interquartile range, IQR) and range. Categorical data is presented as percentages (%). Pearson’s correlation (R) was used to relate SLP derived parameters with volumes measured from spirometry (FVC). Given that we wished to develop a measure for assessing vital capacity measures for children who couldn’t perform spirometry we used Lin’s concordance correlation (*p*c) to determine to what extent SLP derived lung volumes (FVC_SLP_) could be used to replace spirometry values (FVC spirometry). A *p*c value < 0.90 indicates poor strength of agreement. We determined SLP measurement repeatability using the method described by Bland and Altman and the 95% Limits of Agreement [18].

## Results

### Study 1: Relating FVC measurement using SLP with FVC measured using spirometry

Thirteen children and young people with NMD were recruited (Table 1) along with 14 healthy volunteers to compare FVC measured with SLP and with spirometry. The mean age was 12 years (SD = 4.6) for children with NMD and 26 years (SD = 11) for healthy controls.

**Fig 2:**
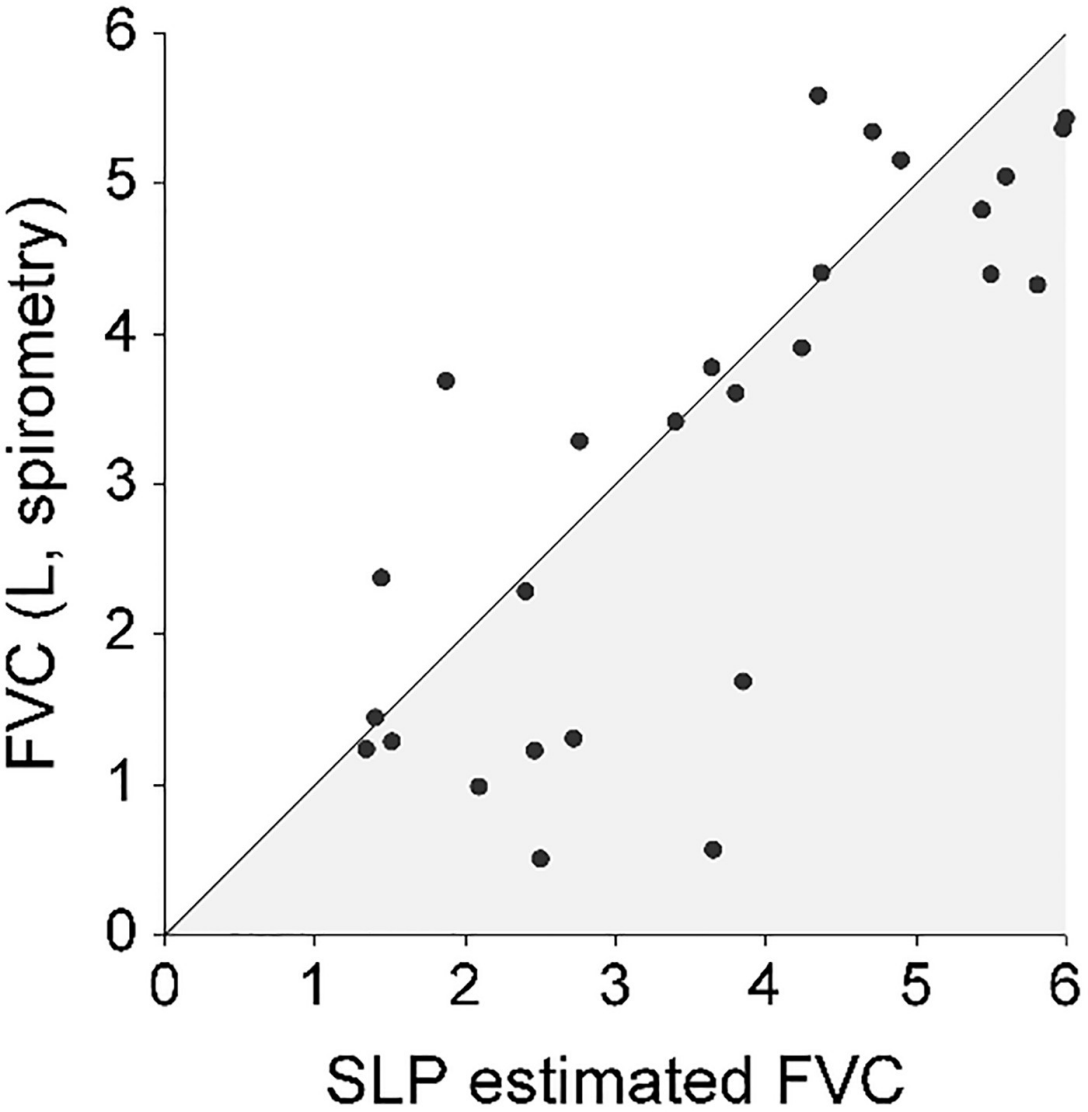
Scatterplot of paired FVC measurements by spirometry and SLP showing the line of identity (perfect agreement). The Pearson’s correlation coefficient was R = 0.78, Lin’s concordance coefficient pc = 0.76.

**Fig 3:**
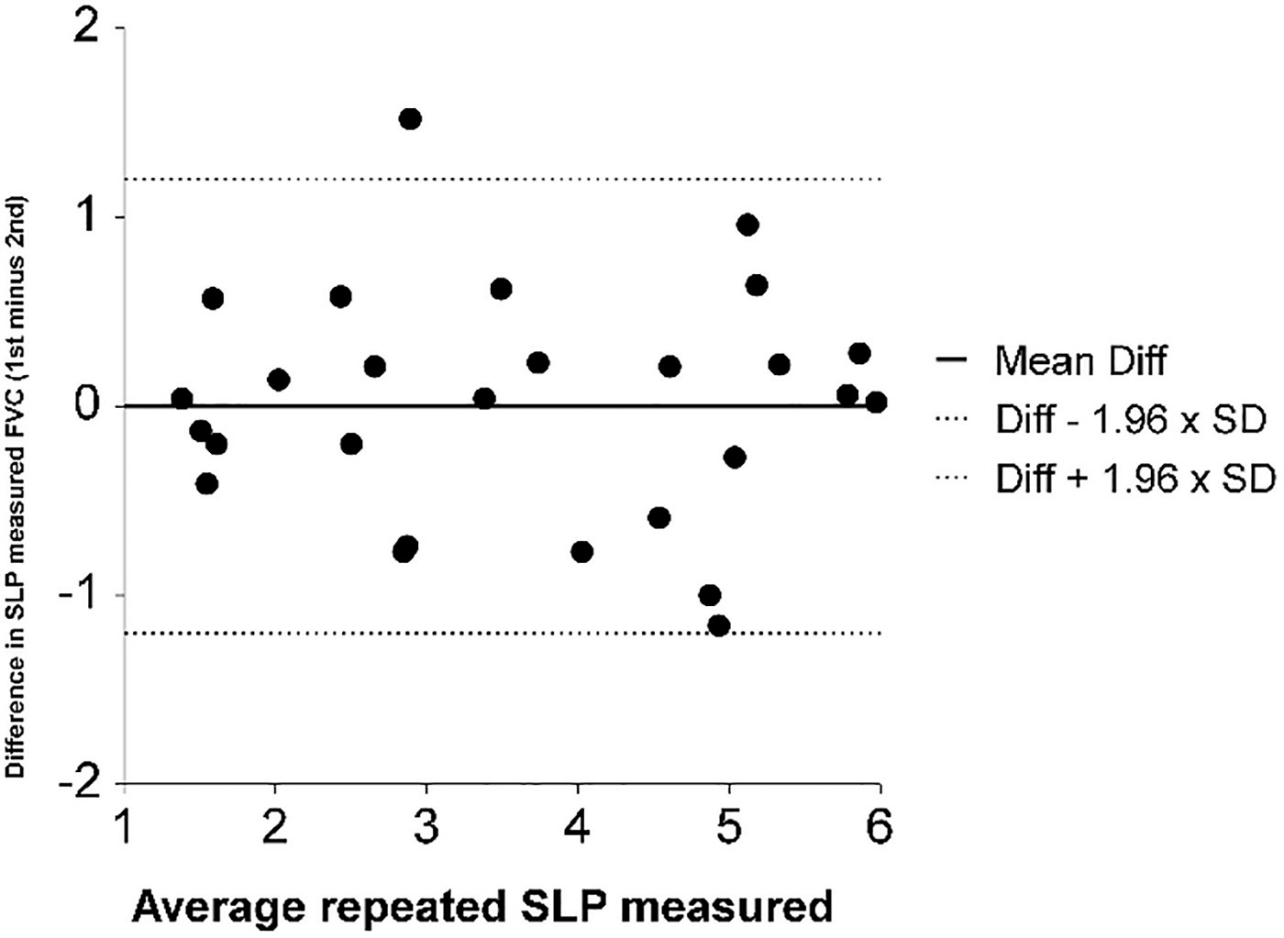
Bland & Altman plot of the difference between the two repeated SLP measurements of FVC (SLP estimated Litres) on the y-axis and the average of the two measures on the x-axis. The mean difference between paired measurements was 0.004 and the 95% Limits of agreement were -1.2 to 1.2 L.

The range of FVC_(spirometry)_ was 0.51 to 5.6 litres and for FVC_(SLP)_ was 1.3 to 6.0 “estimated litres”. Pearson’s correlation between FVC_(SLP)_ and FVC_(spirometry)_ was good at R = 0.78 while Lin’s concordance (pc) was poor at 0.76 (Fig 2). The mean difference between the two FVC_(SLP)_ measurements was 0.004 (SD = 0.61, 95%CI: -0.24 to 0.25) and the 95% Limits of Agreement ranged from -1.2 to 1.2 L (Fig 3).

### Study 2: Using SLP Tidal Breathing parameters to examine the relative contribution (%) of right side and left side thoraco-abdominal wall and the relative contribution (%) of rib cage and abdominal wall

#### Relative contribution of right side and left side TA displacement

**Fig 4:**
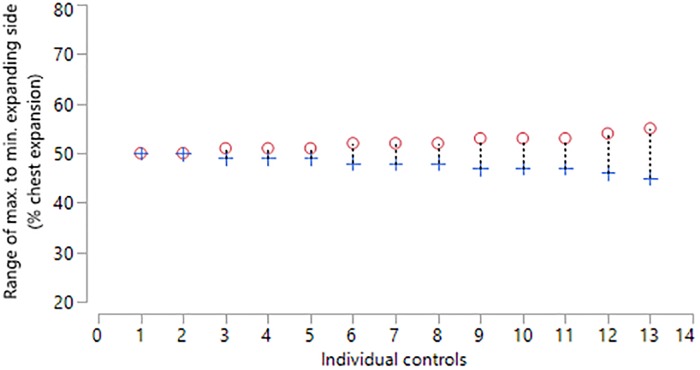
Percentage right and left thoraco-abdominal (TA) contributions for healthy controls (n = 13) during one minute of tidal breathing. Circle = maximal expanding side, Cross = minimal expanding side.

**Fig 5:**
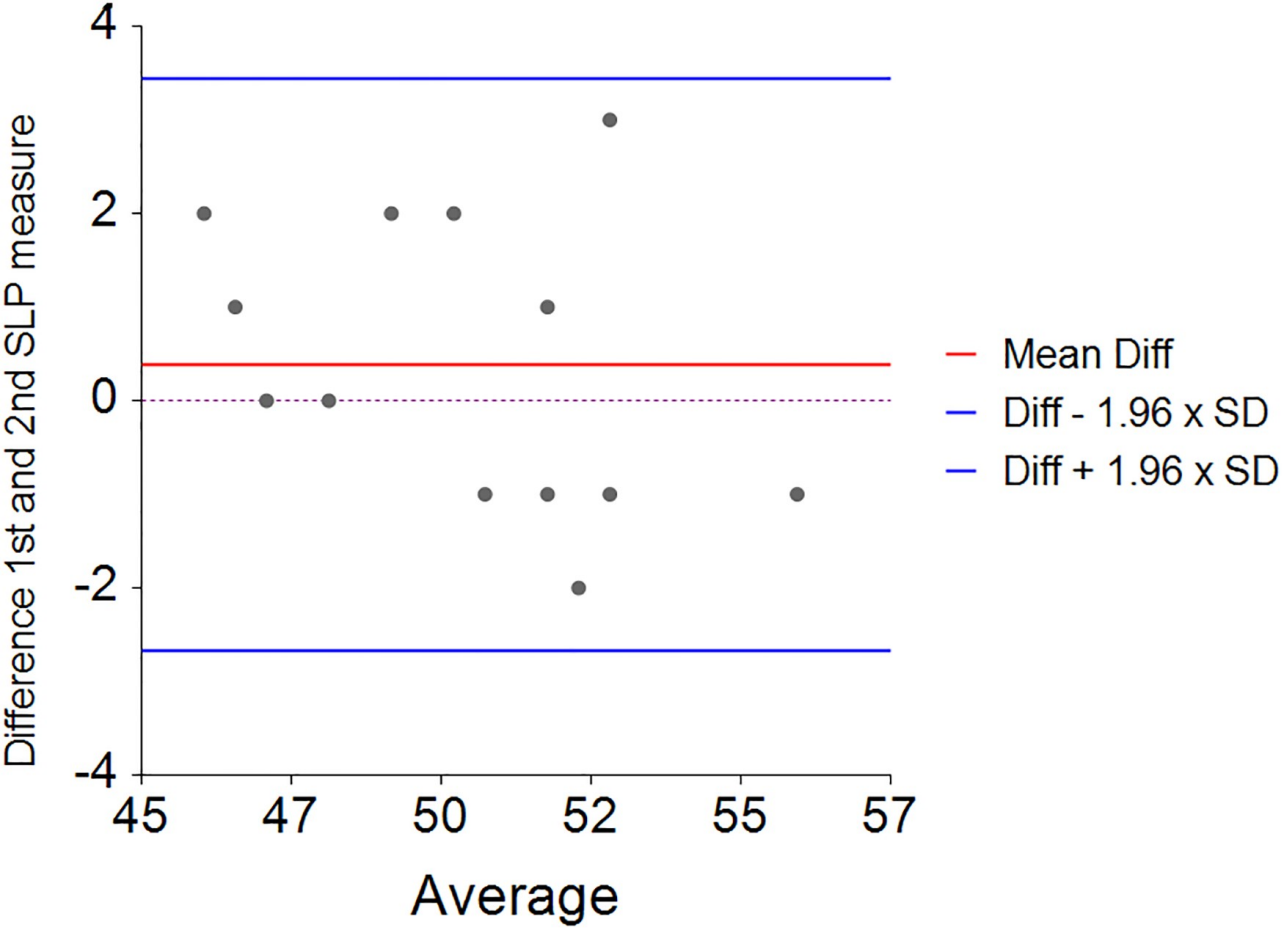
Bland and Altman repeatability plot of tidal breathing (SLP, estimated Litres) differences 1st and 2nd measures for right sided thoraco-abdominal expansion (%) and the average for the control group.

Thirty-four children and young people with NMD (70% with Muscular Dystrophy, mean age 13 years [SD = 3.4]) were recruited (Table 1) and thirteen otherwise healthy adults (hospital staff and students) volunteered, mean age 22 [SD = 1.4]) as a control group. The data is presented separately for the control group and the NMD patients as some of the NMD patients had a scoliosis. For the control group, the mean relative contribution of the right side was 50% (SD = 2.6) indicating that the normal right and left contribution are equal (Fig 4). The mean difference between two consecutive measurements of the right-side percentage contribution was 0.38 percent (SD = 1.45) indicating good repeatability (Fig 5). The 95% limits of agreement for the repeated measures were -2.6 to 3.4 percent.

**Fig 6:**
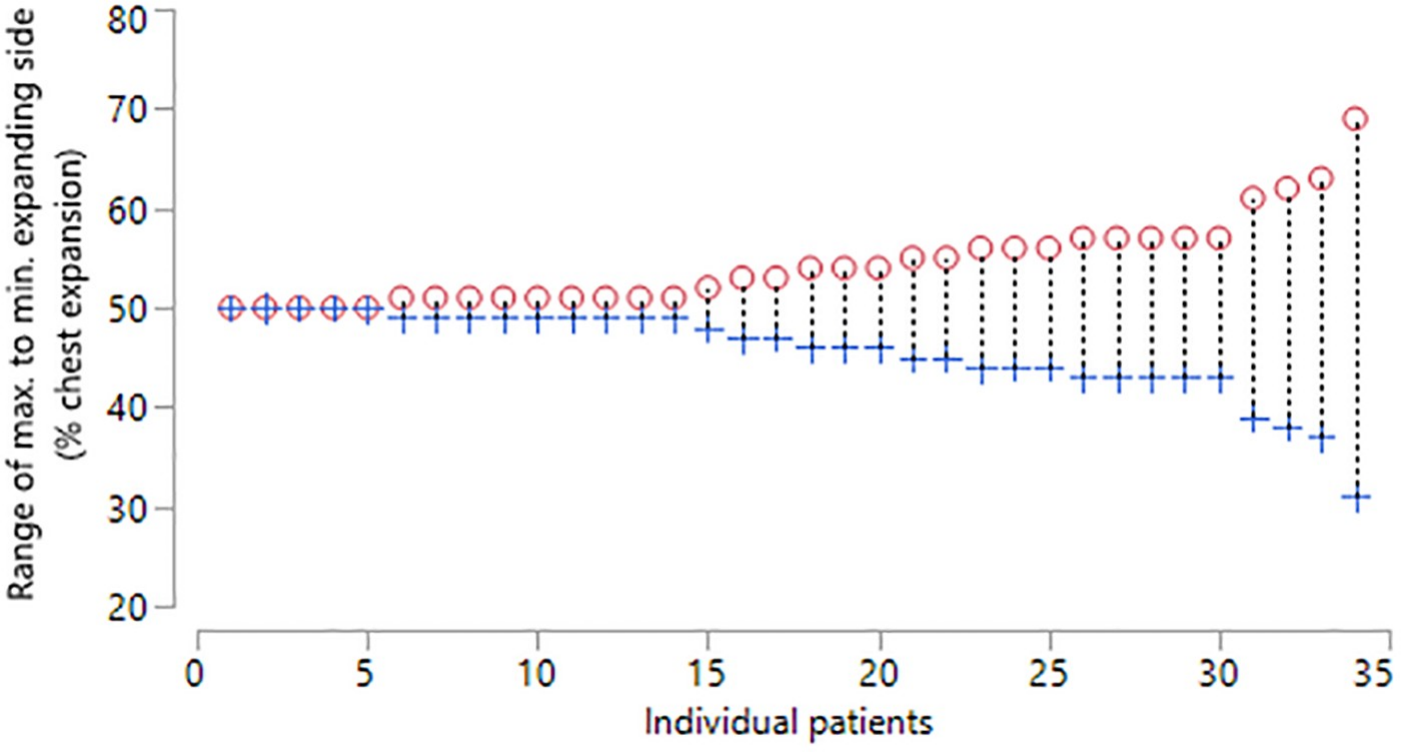
Percentage right and left thoraco-abdominal contributions for patients (n = 34) during one minute of tidal breathing. Circle = maximal expanding side, Cross = minimal expanding side.

**Fig 7:**
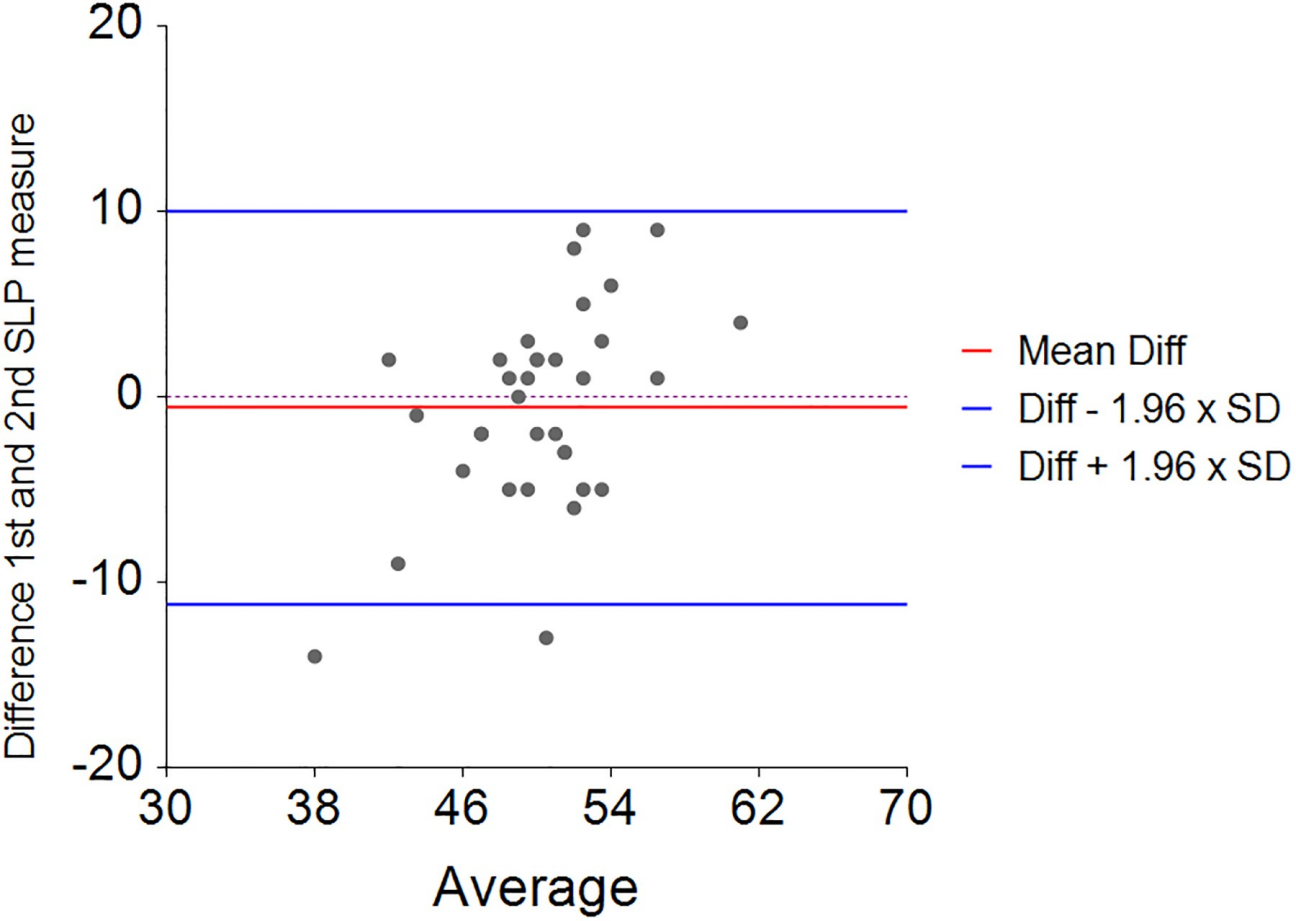
Bland and Altman repeatability plot of tidal breathing (SLP) differences 1st and 2nd measures for maximal expanding side (%) and the average for the patient group.

Right and left contribution in the NMD children are demonstrated in Fig 6. Given that the NMD children could have a scoliosis on either left or right side with an associated lung restriction, the results were reported for the maximal expanding side (MES). The mean relative contribution of the MES was 54% (SD = 4.6) indicating a wider range (range 50% to 69%). The mean difference between two consecutive measurements of the MES percentage contribution was -0.6 percent (SD = 5.4) indicating only moderate repeatability (Fig 7). The 95% limits of agreement for the repeated measures were -11.2 to 10 percent.

#### Relative contribution of rib cage and abdominal wall displacement

**Fig 8:**
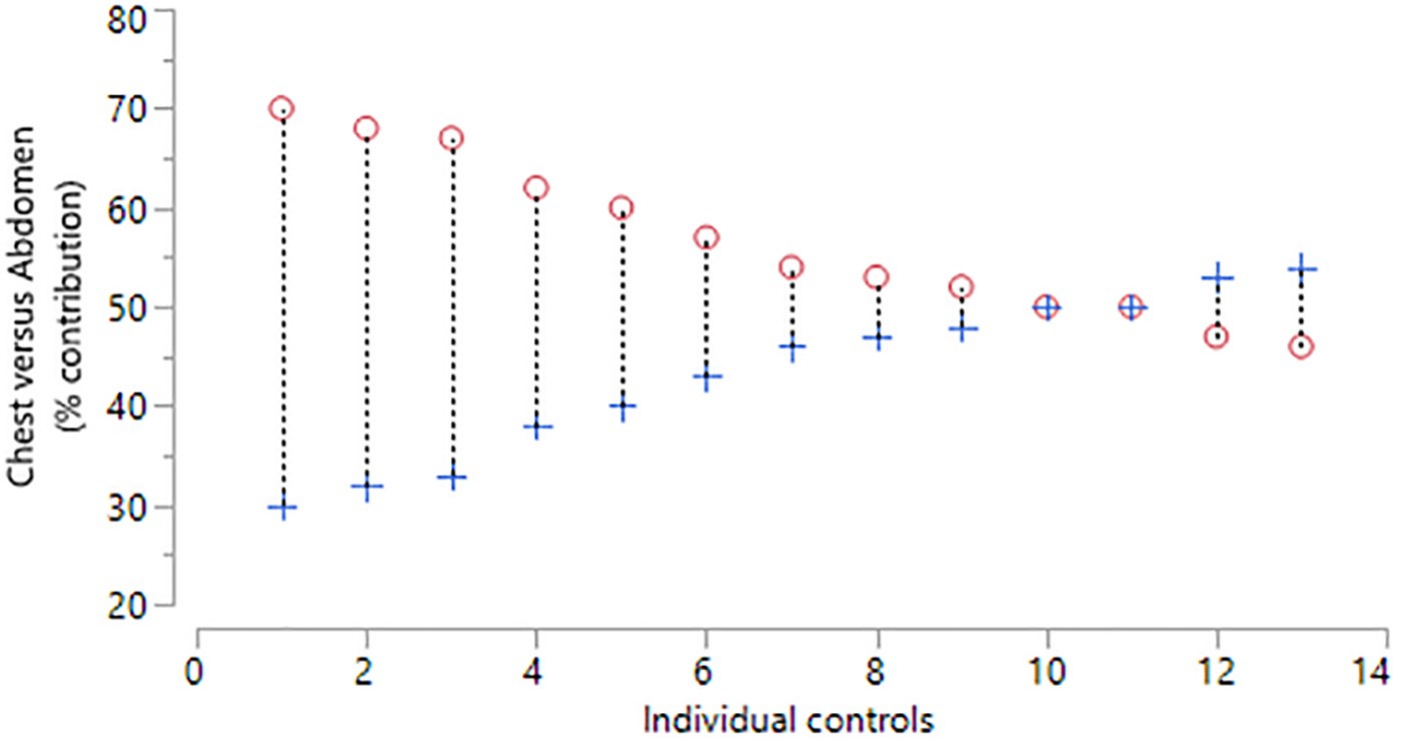
Percentage rib cage and abdominal wall contribution to total TA displacement for controls (n = 13) during one minute of tidal breathing. Circle = Rib cage contribution, Cross = Abdominal wall contribution.

**Fig 9:**
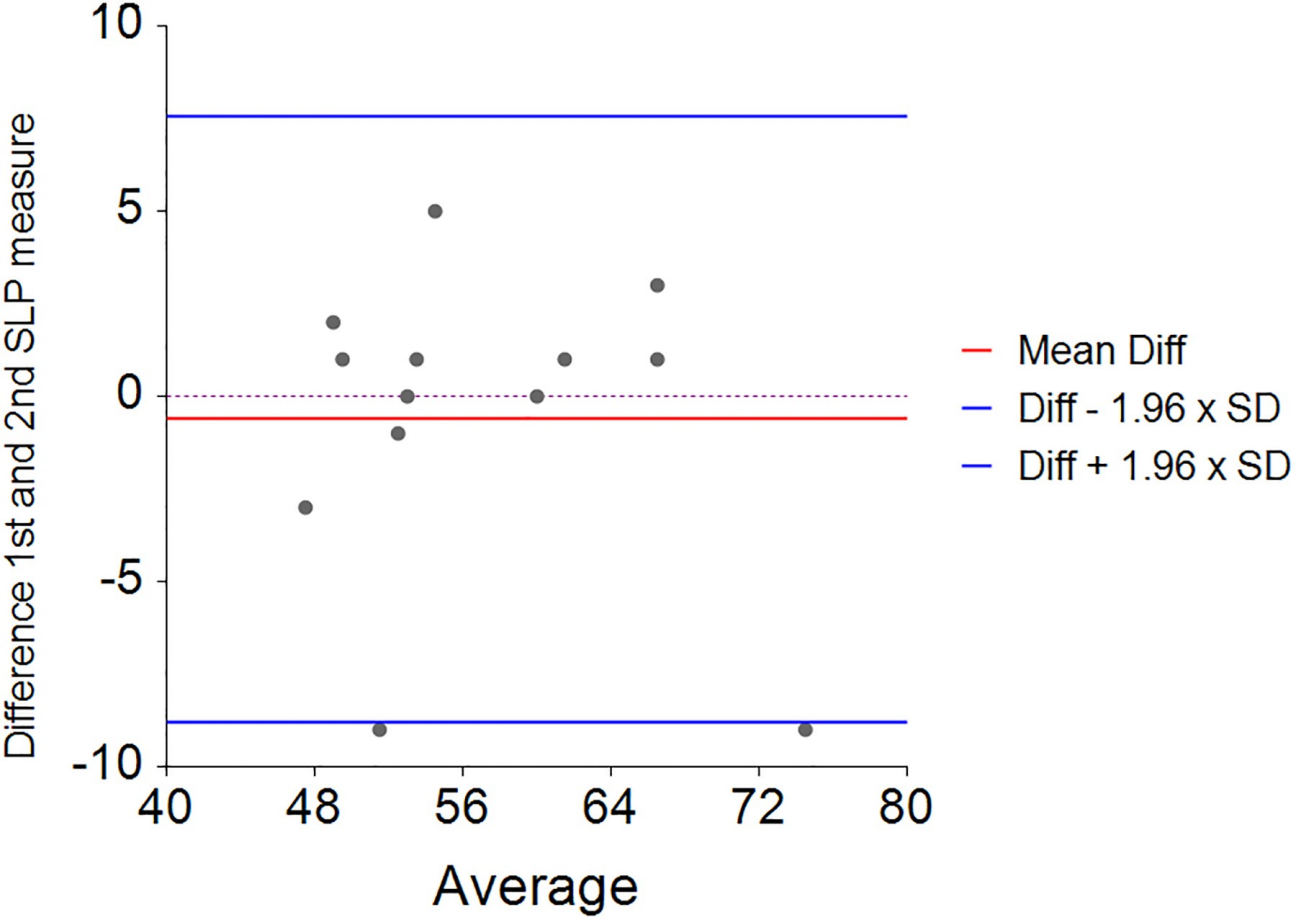
Bland and Altman plot of tidal breathing (SLP); 1st minus 2nd measurement and mean for percentage contribution of thoracic expansion in the control group.

In addition, in the second study, normal healthy volunteers were found to have a mean relative contribution of rib cage displacement of 56% (SD = 8, range 46 to 70) indicating that the normal rib cage displacement and abdominal wall displacement is an average ratio of 56:44 (Fig 8). The mean difference between two consecutive measurements of the rib cage displacement (% contribution) was -0.6 percent (SD = 4.2) indicating moderate repeatability (Fig 9). The 95% limits of agreement for the repeated measures were -8.7 to 7.6 percent.

**Fig 10:**
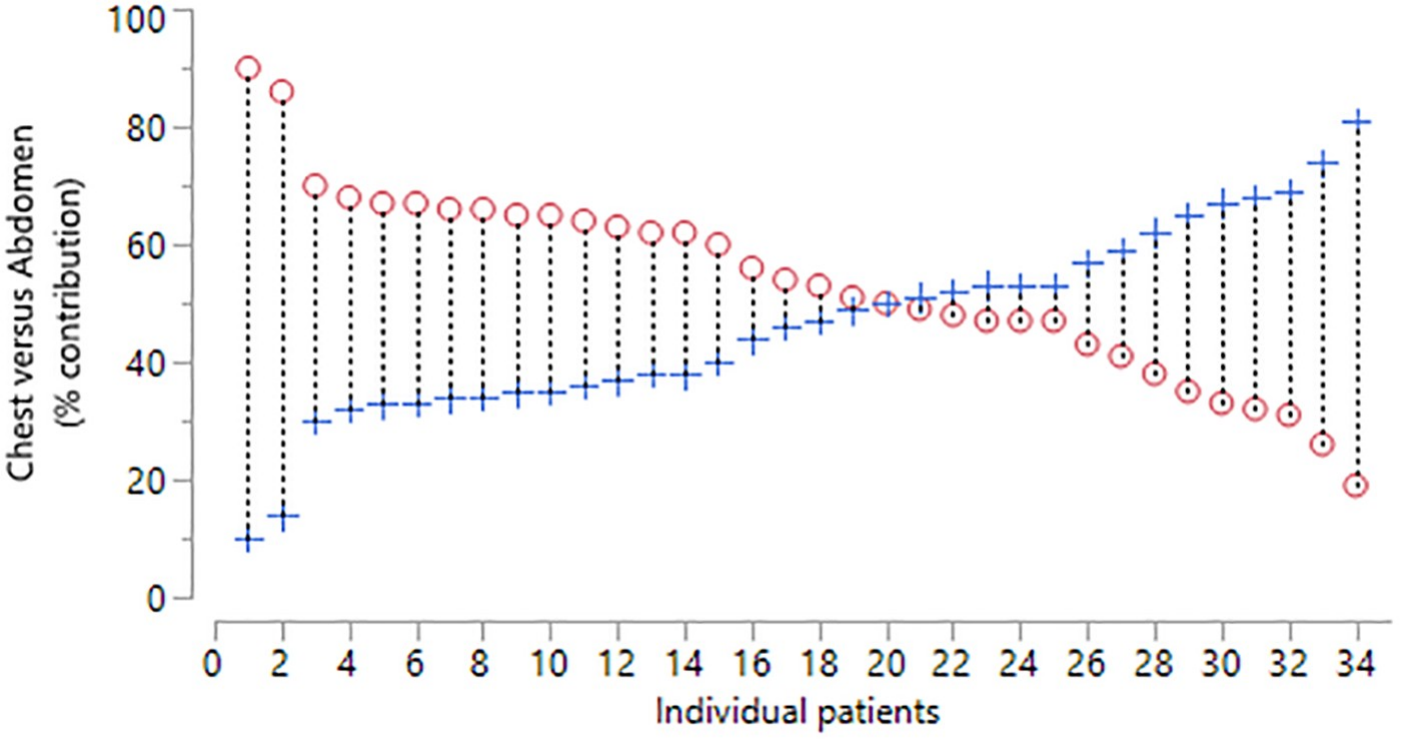
Percentage rib cage and abdominal wall contribution to total TA displacement for patients (n = 34) during one minute of tidal breathing. Circle (o) = rib cage contribution, Cross (+) = abdominal wall contribution.

**Fig 11:**
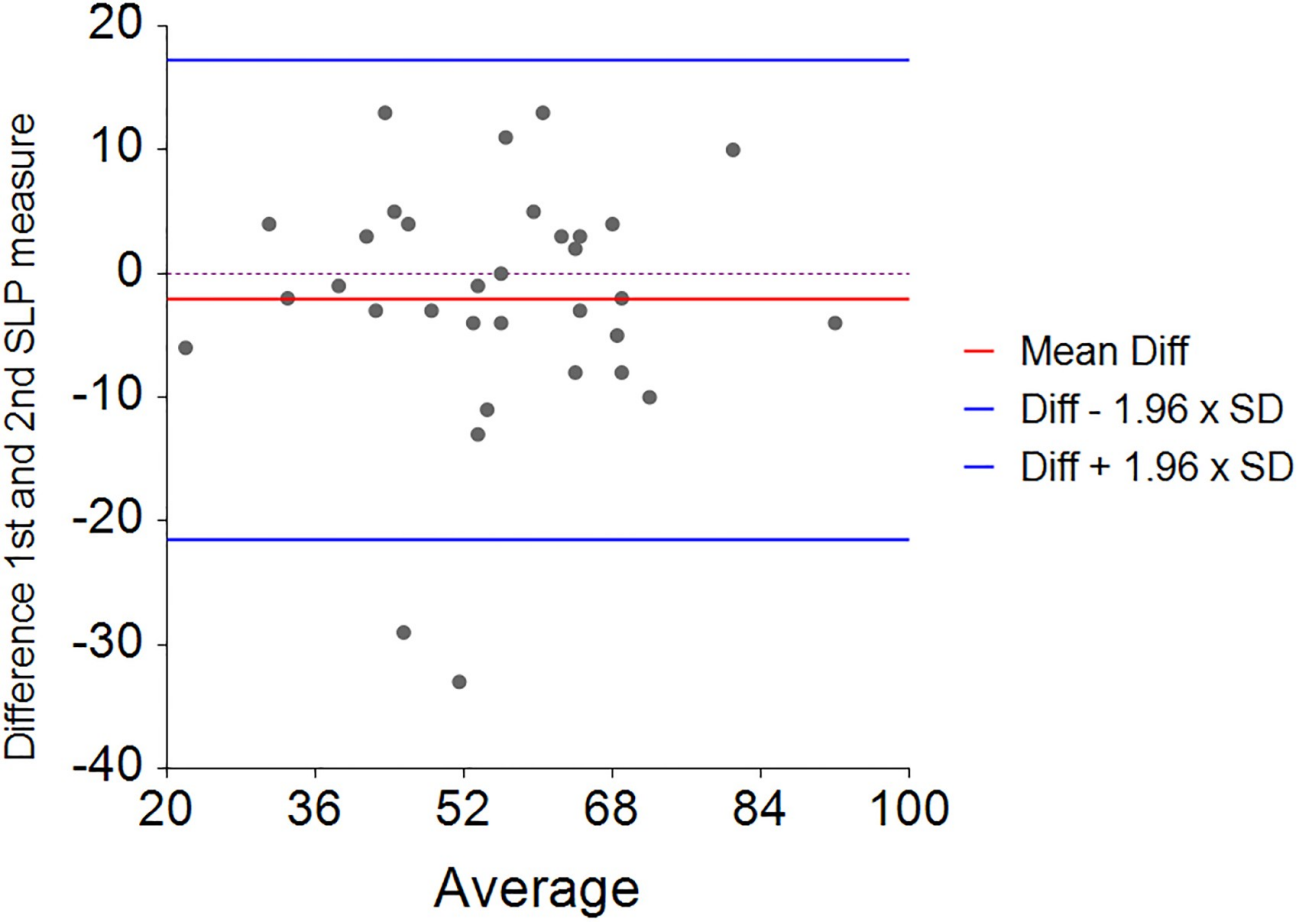
Bland and Altman repeatability plot of tidal breathing (SLP); 1^st^ minus 2nd measurement and average for percentage contribution of thoracic expansion in the patient group.

In those with NMD the mean relative contribution of the rib cage was 54% (SD = 17, range 19 to 90%) indicating a wider range in children with NMD (Fig 10). After removing one outlier with very discrepant repeated measures the mean difference between two consecutive measurements of the rib cage displacement percentage contribution was 2.1 percent (SD = 9.8) indicating moderate repeatability (Fig 11). The 95% limits of agreement for the repeated measures were -21.5 to 17.

**Fig 12:**
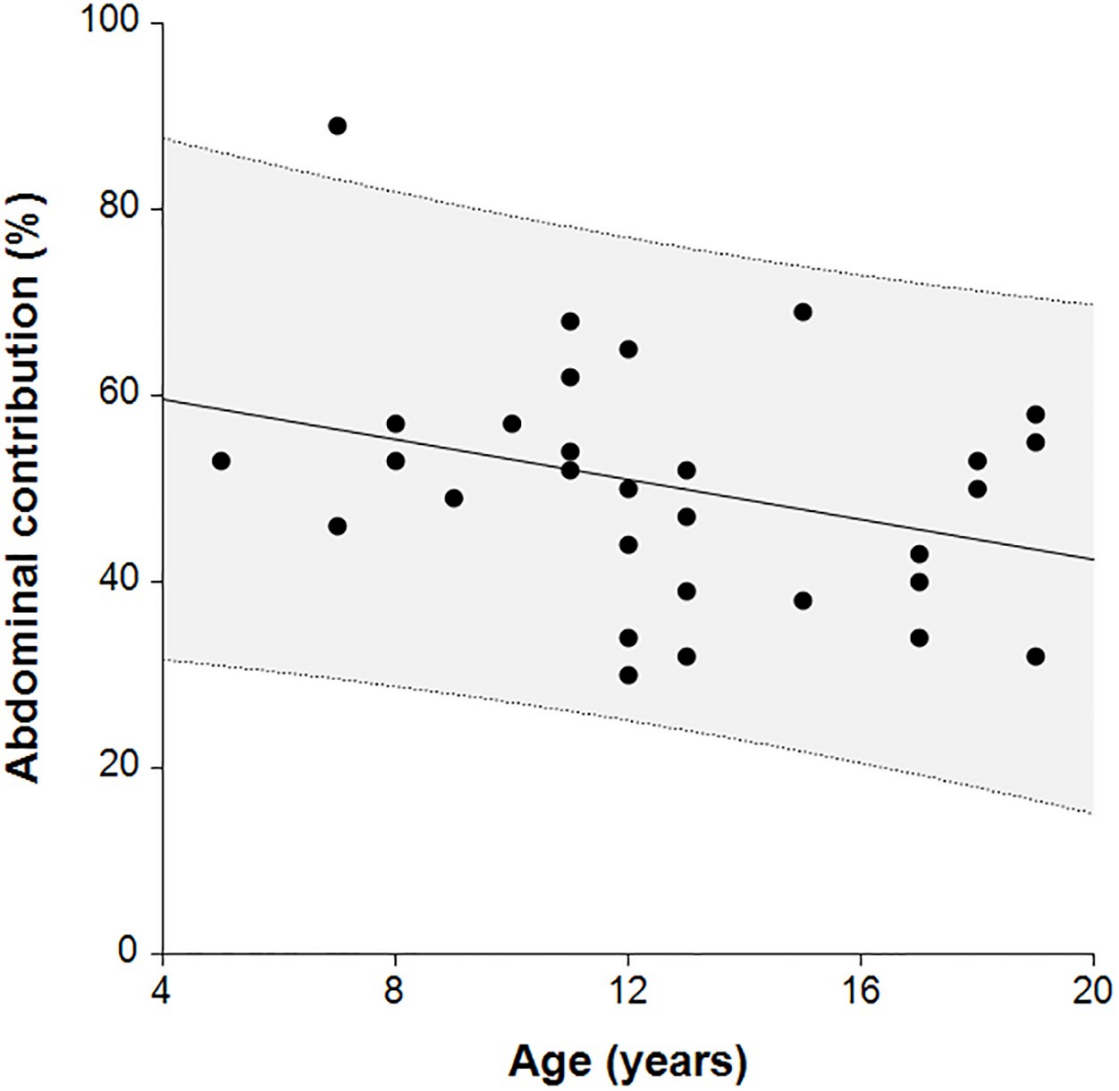
DMD patients reducing abdominal contribution with increasing age.

In DMD patients we found a trend for the abdominal contribution (%) to decrease with increasing age (R = - 0.33, Fig 12).

#### Observed breathing patterns

**Fig 13:**
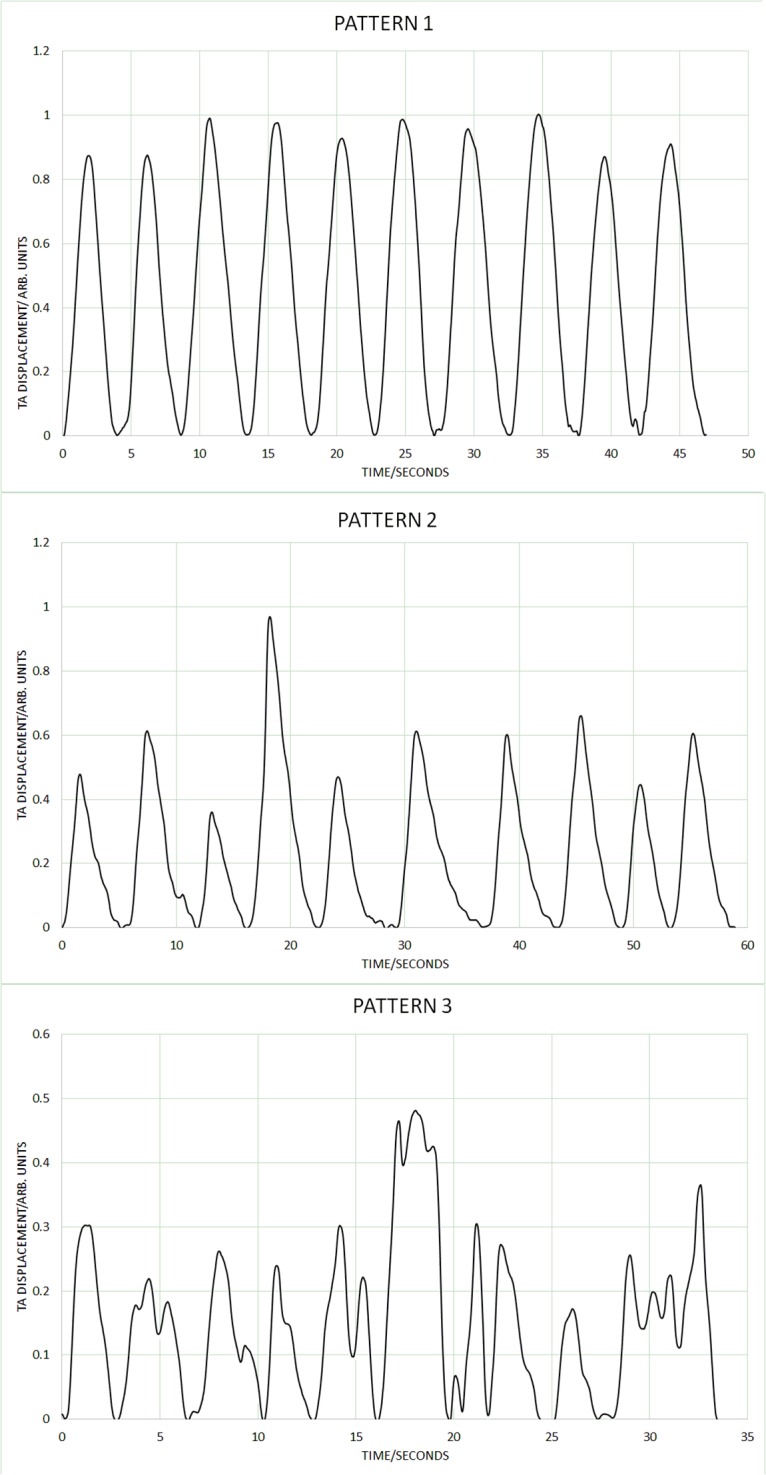
Typical examples of corrected SLP tidal breathing patterns in TA displacement. Pattern 1: regular amplitude and frequency, Pattern 2: regular amplitude and frequency with extended expiratory phase, Pattern 3: irregular amplitude and frequency.

Breathing patterns (graphical representation of the movement of the TA wall) produced with SLP during tidal breathing for both the controls and patients with NMD were labelled according to the system described previously. Three patterns of TA displacement were observed (Fig 13). All control participants demonstrated either pattern 1 or pattern 2. The third pattern was only observed within the NMD group. Of the 34 patients, 11 demonstrated pattern 3, 8 demonstrated pattern 2 and 14 demonstrated pattern 1, with one patient demonstrating a mixture of pattern 2 and 3. All traces were deemed adequate with no SLP equipment software warnings for trace inadequacies. The SLP equipment software is able to identify a poor trace due to a weak signal caused by excessive wrinkles in clothing. The trace displayed on the screen in this case is a mixture of blue and orange rather than the normal blue trace seen for an acceptable recording.

#### Specific illustrative cases for other potential uses for SLP measurements

**Fig 14:**
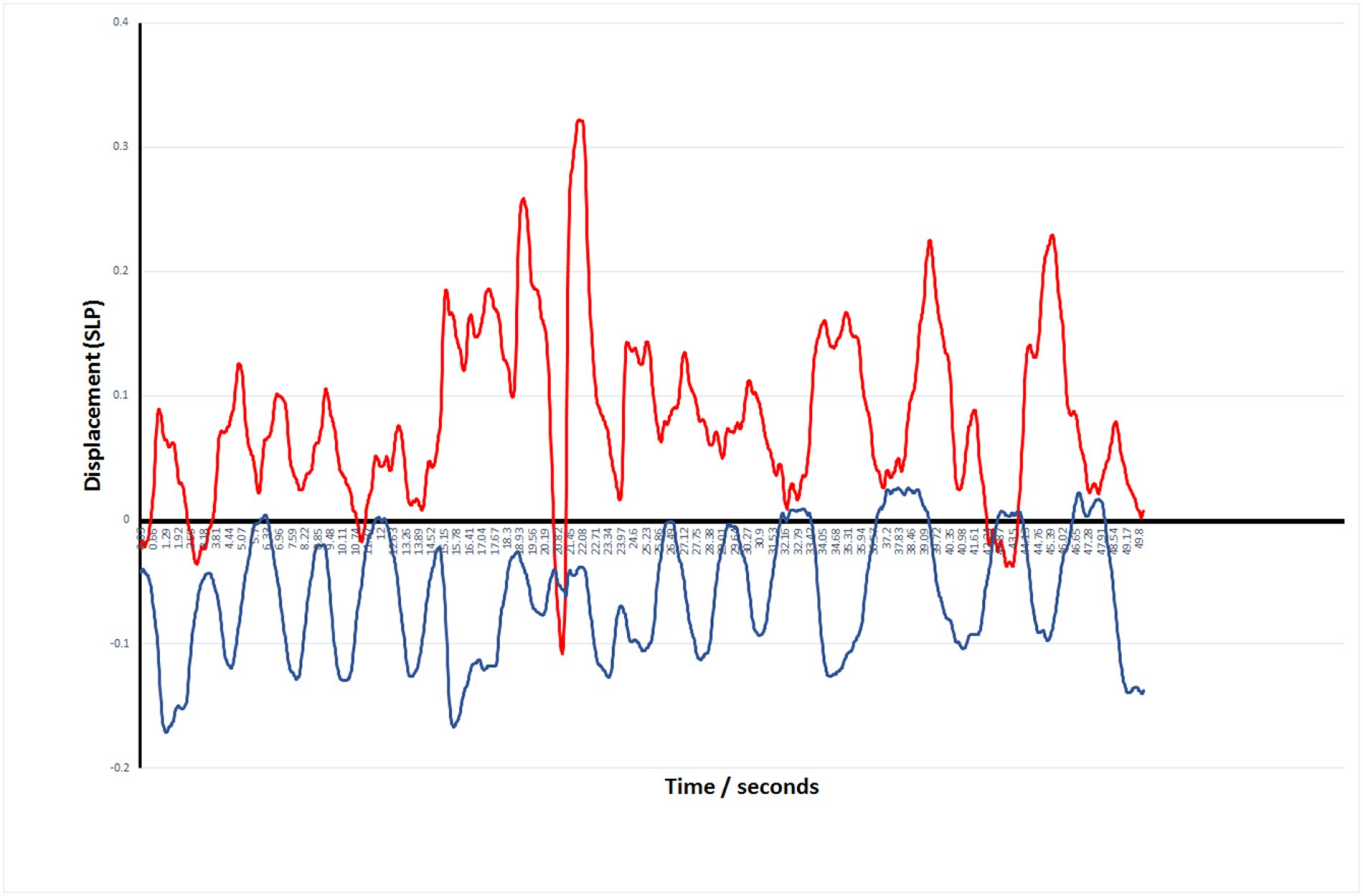
Rib cage and abdominal displacement measured with SLP during 10 breaths for a patient with spinal muscular atrophy (Type 2) showing clear evidence of paradoxical breathing.

Case 1: One boy, aged 13, with spinal muscular atrophy (SMA) displayed regular paradoxical breathing which results from weakness of the intercostal muscles with the diaphragm strength being relatively maintained [19]. This paradoxical breathing can be clearly seen in Fig 14 where the abdominal displacement is out of phase with the rib cage illustrated within the box.

Case 2: One boy, a 19-year-old with DMD and a scoliosis, was assessed with SLP prior to applying commencing Non Invasive Ventilation (NIV) therapy and had SLP repeated while on NIV. Prior to applying NIV his relative contribution of the right and left sides of the TA to total TA displacement was measured at 55:45 respectively and during NIV therapy this was seen to be 50:50. There was no significant difference in the results for the relative contribution of rib cage or abdomen to total TA displacement so it would appear that both lungs were being recruited equally and the diaphragm was not being distended into the abdomen.

## Discussion

The main objective of the studies was to investigate the use of SLP in the clinical environment to assess thoraco-abdominal displacement in children with neuromuscular disorders.

FVC_(SLP)_ compared to FVC_(spirometry)_, had a correlation of r = 0.78, however, these two measurements did not have tight concordance. Furthermore, the values generated by SLP are arbitrary units which do not compare directly to FVC_(spirometry)_ which is measured in litres. Repeated measures of FVC_(SLP)_ demonstrated moderate repeatability. Together these results indicate that SLP measurements could not be used interchangeably with spirometry in the clinical environment. For example, in the case of a child unable to perform conventional spirometry, SLP did not produce a comparable value for FVC. There were several issues encountered when recording SLP data from NMD patients. Firstly, the children had to maintain a vertical sitting position over the recording period. It was observed that older children had greater difficulty in maintaining the required seated position, with some children leaning to one side of the chair during recording. Secondly, some children were inclined to raise their shoulders whilst breathing which may have impacted on the TA wall displacement. This vertical movement of the shoulders may have been due to accessory respiratory muscles being activated during tidal breathing, often seen as a compensatory mechanism when the diaphragm is weak [20]. However, as there was a strong relationship between FVC_(SLP)_ and FVC_(spirometry)_ there may still be a place for SLP as an alternative technology to monitor decline in lung function over time, especially in children with NMD who have difficulty performing spirometry.

The mean relative contribution of right and left TA wall displacement in healthy controls was 50:50 with a relatively narrow range and concurs with the results found by Lanini et al (2003) using opto-electronic plethysmography (OEP) [21]. Although OEP is a reliable method of tracking TA volume displacement, it is less suited to the clinical environment. Repeatability of this measure with SLP was found to be good in healthy controls and moderate in NMD children with or without scoliosis but with a wider range. The moderate repeatability in NMD may be caused by the variable breathing patterns observed in some NMD children. However, this would require further investigation in a well-defined cohort. The child with the most severe scoliosis displayed 31% right side contribution and 69% left side contribution. Further investigation is required to ascertain how the Cobb angle of a scoliosis correlates with the relative contribution of right and left sides. Currently, there is no method of assessing the relative contribution of right and left sides to lung function before and after corrective scoliosis surgery. Therefore, SLP may provide useful clinical information for these patients. In addition, SLP may prove invaluable when NIV or cough assist is being introduced. SLP may help optimise the pressure and volume settings to ensure maximum expansion of the TA wall.

The mean relative contribution of rib cage and abdominal wall in the seated position for healthy controls was 59:41 with moderate repeatability. The corresponding mean for children with NMD was 54:46 with a wider range and with reduced repeatability. Reduced repeatability of this measure in NMD children may be due to the weaker respiratory muscles which are not able to move the TA wall in the same way with each breath. If the contribution of the accessory respiratory muscles to each breath varies then the movement of different regions of the TA wall may be affected resulting in non-repeatable SLP displacement measurements. Most of the control group displayed a predominant rib cage displacement during tidal breathing (Fig 10) and those who displayed predominant abdominal wall displacement showed displacement of both regions close to 50:50. In comparison, children with NMD were more likely to display a higher contribution of abdominal wall displacement (n = 14). The range of displacement of the TA wall in the NMD group was higher than the control group; the highest abdominal wall contribution to TA wall displacement was 81% and the lowest was 10%. A range of respiratory muscles are impacted to varying degrees within different forms of NMD. This may explain the wide range of mean relative contributions and it may be advisable to study these clinical sub-groups separately. When participants with DMD were analysed separately it was found that the abdominal contribution to total TA displacement reduced with age. This was observed by Lo Mauro et al (2018) with OEP in a study of 115 DMD children [22]. This reduction in abdominal displacement may reflect the weakening of the diaphragm as DMD progresses [23]. It is also worth noting that the DMD patient with the lowest abdominal contribution to tidal inspiration (17.4%) was the only DMD patient currently receiving nocturnal NIV therapy and was also the eldest DMD patient at 19 years, suggesting that this SLP measurement may identify the optimal time for introduction of nocturnal NIV. A 13-year-old child with SMA, which is known to cause weakness of the intercostal muscles whilst diaphragmatic strength is relatively maintained [23], displayed a clear paradoxical breathing pattern (Fig 14). Inspiration and expiration phases are not clearly identifiable in Fig 14 therefore further investigation with SLP to diagnose diaphragmatic or intercostal muscle weakness is required. To clearly discriminate between intercostal and diaphragmatic muscle weakness the SLP recording would need to be co-ordinated accurately with clinician directed breathing. It is worth noting that this participant displayed the lowest relative contribution of the rib cage to total TA displacement which agrees with observations made by Perez et al (1996), using respiratory inductive plethysmography, when they found that the relative contribution of the rib cage was low in patients with SMA [24].

SLP recording from 27 controls and 47 children with varying forms of NMD demonstrated three clear patterns of breathing. To our knowledge these patterns have not previously been reported in the literature. SLP was excellent at demonstrating very small changes in TA wall motion for both the inspiratory and expiratory phases. Both controls and children with NMD demonstrated pattern 1 in which the inspiratory and expiratory phases were symmetrical in terms of duration. However, the expiratory phase in pattern 2 was of longer duration than the inspiratory phase which may indicate obstructive breathing. Pattern 3 was observed in 11 of the 34 participants in the NMD group and none of the control groups. Further investigation is required to monitor breathing using SLP as NMD progresses to assess the relevance and importance of these patterns.

### Limitations

There were a few limitations within the studies. Firstly, the control group was made up of adults who did not display any obvious lung disease recruited from staff and students within the hospital. However, it would have been more appropriate to have age-matched children as controls.

We obtained data regarding the mean relative contribution of right and left TA wall displacement in 76 healthy normal children and young people aged between 5 and 21 years from Pneumacare (www.pneumacare.com). This confirmed that the right versus left chest wall movement was 50:50 similar to our findings in older controls. However, the mean relative contribution of rib cage and abdominal wall for their healthy controls was 53:47 much more closely in line with our patient group’s 54:46 and different from our older healthy controls (59:41). This unpublished data provided by Pneumacare also suggested that over the full life course the mean relative contribution of rib cage and abdominal wall changed with aging with an increased relative contribution from chest wall movement. Clearly it will be important for the normative values and how they change with age and gender to be formally studied.

Secondly, we had a limited number of patients (n = 4) who had been diagnosed with a scoliosis. It would have been beneficial to have a greater number of patients with a range of Cobb angles to assess the SLP measurements of TA displacement. Thirdly, the head of the SLP system can be raised and tilted to ensure the projected grid pattern covers the whole of the TA wall. No assessment was made of the positioning of the head or potential impact on TA wall displacement measurements. Further investigation is required to ascertain if this tilt, which affects the angle at which the light beam reaches the TA wall, has any impact on results. Fourthly, only two SLP measurements were made when comparing spirometry with SLP, whilst up to 8 spirometry measurements were acquired (as per BTS guidelines) to ensure an accurate measurement is obtained. SLP results may have demonstrated better repeatability and accuracy if more attempts had been made and the best SLP results compared with the best spirometry results. Finally, with regards clothing, wrinkles on the T-shirt during SLP measurement were often an issue and may have affected the ability to obtain accurate SLP measurements.

## Conclusion

To our knowledge these are the first studies using SLP to assess thoraco-abdominal breathing patterns in NMD. Our studies have shown that SLP cannot be used interchangeably with spirometry to assess forced expiratory flows and volumes. However, SLP is able to assess the relative contribution of the right and left sides of the TA wall to the total TA displacement in healthy individuals and in children with neuromuscular disease and can be used simultaneously with NIV therapy. It may therefore have a role to play in monitoring respiratory function in children with neuromuscular disease over time and may help to optimise interventions for this patient population. SLP identified three specific patterns of breathing amongst healthy individuals and in children with NMD. Further research is required to assess the significance of these patterns. It is still relatively early in the development of SLP technology and the analysis of lung function parameters that it is able to collect and measure. Improved repeatability through clinician/physiologist training and patient engagement will improve its diagnostic capabilities.

## References

[1] Hull J, Aniapravan R, Chan E, Chatwin M, Forton J, Gallagher J, et al British Thoracic Society guideline for respiratory management of children with neuromuscular weakness. *Thorax*. 2012;67(Suppl 1): 1–40. 22730428 10.1136/thoraxjnl-2012-201964

[2] Howard RS, Wiles CM, Hirsch NP, Spencer GT. Respiratory involvement in primary muscle disorders: assessment and management. *Quarterly Medical Journal*. 1993;86(3): 175–189. 8483991

[3] Bach J.R., Ishikawa Y., Kim H. Prevention of pulmonary morbidity for patients with Duchenne muscular dystrophy. *Chest*. 1997;112: 1024–1028 10.1378/chest.112.4.1024 9377912

[4] Gozal D. Pulmonary manifestations of neuromuscular disease with special reference to Duchenne muscular dystrophy and spinal muscular atrophy *Pediatric Pulmonology*. 2000;29: 141–150. 10639205 10.1002/(sici)1099-0496(200002)29:2<141::aid-ppul9>3.0.co;2-y

[5] Fauroux B, Quijano-Roy S, Desguerre I, Khirani S. The value of respiratory muscle testing in children with neuromuscular disease. *Chest Journal*. 2015;147(2): 552–559. 10.1378/chest.14-0819 25644908

[6] Phillips MG, Quinlivan RCM, Edwards RHT, Calverley PMA. Changes in spirometry over time as a prognostic marker in patients with Duchenne Muscular Dystrophy. Amer J Resp Crit Care Medicine, 2001; 164(12): 2191–2194 10.1164/ajrccm.164.12.2103052 11751186

[7] Fauroux B, Khirani S. Neuromuscular disease and respiratory physiology in children: putting lung function into perspective. *Respirology*. 2014;19(6): 782–791. 10.1111/resp.12330 24975704

[8] Ricotti V, Mandy WPL, Scoto M, Pane M, Deconinck N, Messina S, Mercuri E, Skuse DH, Muntoni F. Neurodevelopmental, emotional, and behavioural problems in Duchenne muscular dystrophy in relation to underlying dystrophin gene mutations. *Development Medicine and Child Neurology*. 2015;58(1): 77–84. 10.1111/dmcn.12922 26365034

[9] Perez T. Neuromuscular disorders—assessment of the respiratory muscles. *Rev Neurol (Paris)*. 2006;162(4): 437–444. 16585904 10.1016/s0035-3787(06)75034-2

[10] Gauld LM, Kappers J, Carlin JB, Robertson CF. Prediction of Childhood Pulmonary Function Using Ulna Length. *American Journal of Respiratory Critical Care Medicine*. 2003;168: 804–809. 10.1164/rccm.200303-451OC 12869362

[11] Schroth MK. Special considerations in the respiratory management of spinal muscular atrophy. *Pediatrics*. 2009;123(4): S245–9. 19420154 10.1542/peds.2008-2952K

[12] Alan J. Pulmonary Complications of Neuromuscular disease: A respiratory mechanics perspective. *Paediatric Respiratory Reviews*. 2010;11: 18–23. 10.1016/j.prrv.2009.10.002 20113987

[13] de Boer W, Lasenby J, Cameron J, Wareham R, Ahmad S, Roach C, Hills W, Iles R. SLP: A zero-contact non-invasive method for pulmonary function testing. British Machine Vison Conference Proceedings 2010.

[14] Lau E, Brand D, Bridge P, Iles R, Wareham RJ, Cameron JI, Usher Smith J, Hills W, Roberts G, de Boer W, Lasenby J. Comparison of forced expiratory volumes measured with structured light plethysmography (SLP) and spirometry. British Thoracic Society Winter Meeting. 2009;12th Feb 2009, London, UK.

[15] Motamedi-Fakhr S, Iles R, Barney A, De Boer W, Conlon J, Khalid A, Wilson RC. Evaluation of the agreement of tidal breathing parameters measures simultaneously using pneumotachography and structured light plethysmography. *Physiological Reports*. 2017;5(3): e13124 10.14814/phy2.13124 28193785 PMC5309576

[16] Alimohamed S, Prosser K, Weerasuriya C, Iles R, Cameron J, Lasenby J, et al P134 Validating structured light plethysmography (SLP) as a non-invasive method of measuring lung function when compared to Spirometry. *Thorax*. 2011;66(4): A121–A121.

[17] Miller MR, Hankinson J, Brusasco V, Burgos F, Casaburi R, Coates A, et al Standardisation of spirometry. *European Respiratory Journal*. 2005;26(2): 319–338. 10.1183/09031936.05.00034805 16055882

[18] Bland JM, Altman DG. Statistical methods for assessing agreement between measurement. Biochimica Clinica 1987;11: 399–404.

[19] Schroth MK. Special considerations in the respiratory management of spinal muscular atrophy. *Pediatrics*. 2009;123: S245–S249. 10.1542/peds.2008-2952K 19420154

[20] Lo Mauro A, Aliverti A. Physiology of respiratory disturbances in muscular dystrophies. *Breathe*. 2016;12: 318–327. 10.1183/20734735.012716 28210319 PMC5297947

[21] Lanini B, Bianchi R, Romagnoli I, Coli C, Binazzi B, Gigliotti F, Pizzi A, Grippo A, Scano G. Chest wall kinematics in patients with hemiplegia. *American Journal of Respiratory Critical Care Medicine*. 2003;168: 109–113. 10.1164/rccm.200207-745OC 12714347

[22] Lo Mauro A, Romei M, Gandossini S, Pascuzzo R, Vantini S, D’angelo MG, Aliverti A. Evolution of respiratory function in Duchenne muscular dystrophy from childhood to adulthood. *European Respiratory Journal*. 2018;51: 1701418 10.1183/13993003.01418-2017 29437939

[23] Lo Mauro A, D’angelo MG, Romei M, Motta F, Colombo D, Comi GP, Pedotti A, Marchi E, Turconi AC, Bresolin N, Aliverti A. Abdominal volume contribution to tidal volume as an early indicator of respiratory impairment in Duchenne muscular dystrophy. *European Respiratory Journal*. 2010;35: 5. 10.1183/09031936.00037209 19840972

[24] Perez A, Mulot R, Vardon G, Barois A, Gallego J. Thoracoabdominal pattern of breathing in neuromuscular disorders. *Chest*. 1996;110: 454–461. 10.1378/chest.110.2.454 8697851

